# HSV-1 cellular model reveals links between aggresome formation and early step of Alzheimer’s disease

**DOI:** 10.1038/s41398-023-02376-8

**Published:** 2023-03-10

**Authors:** Marie Alexandra Albaret, Julien Textoris, Bastien Dalzon, Jérémy Lambert, Morgane Linard, Catherine Helmer, Sabine Hacot, Sandra E. Ghayad, Martial Ferréol, Hichem C. Mertani, Jean-Jacques Diaz

**Affiliations:** 1 Univ Lyon, Université Claude Bernard Lyon 1, INSERM U1052, CNRS UMR5286, Centre Léon Bérard, Cancer Research Center of Lyon, 69008 Lyon, France; 2 Department of Translational Research and Innovation, Centre Léon Bérard, 69373 Lyon, France; 3 EA7426, Joint Research Unit, bioMerieux-HCL-UCBL1, hôpital Edouard Herriot 5 place d’Arsonval, 69437 Lyon, France; 4 Chemistry and Biology of Metals, Univ. Grenoble Alpes, CNRS UMR5249, CEA, IRIG, CBM- ProMIT, 17 Avenue des Martyrs, F-38054 Grenoble Cedex 9, Lyon, France; 5 University of Bordeaux, Inserm, Bordeaux Population Health Research Center, UMR U1219, F- 33000 Bordeaux, France; 6 Center for CardioVascular and Nutrition Research (C2VN), INSERM 1263, INRAE 1260, Aix-Marseille University, Faculty of Pharmacy, Marseille, France; 7 INRAE, Ecoflows, UR RiverLy, BP 32108, 69616, 5 Rue de la Doua, 69100 Villeurbanne, France; 8 Institut Convergence PLAsCAN, 69373 cedex 08 Lyon, France; 9 DevWeCan Labex Laboratory, 69373 cedex 08 Lyon, France

**Keywords:** Molecular neuroscience, Pharmacology

## Abstract

Many studies highlight the potential link between the chronic degenerative Alzheimer’s disease and the infection by the herpes simplex virus type-1 (HSV-1). However, the molecular mechanisms making possible this HSV-1-dependent process remain to be understood. Using neuronal cells expressing the wild type form of amyloid precursor protein (APP) infected by HSV-1, we characterized a representative cellular model of the early stage of the sporadic form of the disease and unraveled a molecular mechanism sustaining this HSV-1- Alzheimer’s disease interplay. Here, we show that HSV-1 induces caspase-dependent production of the 42 amino-acid long amyloid peptide (Aβ42) oligomers followed by their accumulation in neuronal cells. Aβ42 oligomers and activated caspase 3 (casp3A) concentrate into intracytoplasmic structures observed in Alzheimer’s disease neuronal cells called aggresomes. This casp3A accumulation in aggresomes during HSV-1 infection limits the execution of apoptosis until its term, similarly to an abortosis-like event occurring in Alzheimer’s disease neuronal cells patients. Indeed, this particular HSV-1 driven cellular context, representative of early stages of the disease, sustains a failed apoptosis mechanism that could explain the chronic amplification of Aβ42 production characteristic of Alzheimer’s disease patients. Finally, we show that combination of flurbiprofen, a non-steroidal anti-inflammatory drug (NSAID), with caspase inhibitor reduced drastically HSV-1-induced Aβ42 oligomers production. This provided mechanistic insights supporting the conclusion of clinical trials showing that NSAIDs reduced Alzheimer’s disease incidence in early stage of the disease. Therefore, from our study we propose that caspase-dependent production of Aβ42 oligomers together with the abortosis-like event represents a vicious circle in early Alzheimer’s disease stages leading to a chronic amplification of Aβ42 oligomers that contributes to the establishment of degenerative disorder like Alzheimer’s disease in patients infected by HSV-1. Interestingly this process could be targeted by an association of NSAID with caspase inhibitors.

## Introduction

Epidemiological and molecular studies [1–4] support the hypothesis that HSV-1, a neurotropic virus that may infect the central nervous system (CNS) chronically, could act as an etiologic co-factor of the neurodegenerative Alzheimer’s disease process. HSV-1 is recognized as a virus targeting essentially the peripheral nervous system. However, epidemiological and molecular studies support the notion of that the chronic infection of the CNS by HSV-1, with the occurrence of reactivation cycles similar to those observed in the peripheral nervous system, provides a biological link between HSV-1 chronic infection and Alzheimer’s disease [5, 6]. HSV-1 LAT mRNA involved in viral latency has been detected in 81 % of Alzheimer’s disease brain patients’ and in the brain of 47 % of healthy aged people [7]. IgG against HSV-1 antigens have been found in the cerebrospinal fluid of 52 % of Alzheimer’s disease patients and 69% of healthy aged people [8]. Individuals homozygotes for the ε4 allele of the ApoE gene exhibit a dramatic increase of risk to develop Alzheimer’s disease when associated with detection of either HSV-1 in the CNS [9] or anti-HSV antibodies in the blood [10]. A mouse model of HSV-1 recurrent infection allowed to point out an accumulation of Alzheimer’s disease hallmarks in the brain of infected mice concomitant to a cognitive decline [11].

A plethora of studies unraveled the dysregulation of Amyloid Protein Precursor (APP) as one of the most validated hallmarks of Alzheimer’s disease. In human brain, APP processing leads to several well characterized peptides, the Aβ family. One of these peptides, Aβ42 is neurotoxic and is found accumulated in the extracellular amyloid plaques characteristic of Alzheimer’s disease brain patients [12]. An interplay between HSV-1 and APP processing has been demonstrated. HSV-1 contributes to the accumulation of Aβ42 [13]. HSV-1 also induces accumulation of a specific HSV-1 C-terminal 55 kDa APP fragment containing Aβ [14], or of APP fragments containing a portion of Aβ [15].

Familial forms of Alzheimer’s disease representing 5% of the patients are associated to different type of APP gene mutations [16]. Many experimental models, genetically-driven, have been developed to analyze gene expression regulation of the mutated forms allowing to decipher the molecular mechanism underlying the impact of these APP mutated forms and the disease [17]. However, the sporadic forms of Alzheimer’s disease accounting for 95% of the patients do not involve mutations in the APP genes. Experimental models allowing to analyze the dysregulation of the APP wild type (WT) gene processing and its potential links with the disease are missing.

Here, we report a model of neuronal cells infected by HSV-1 expressing WT APP in which caspase-dependent production of Aβ42 oligomers followed by their accumulation in neuronal cells occur. Aβ42 oligomers and casp3A concentrate into aggresomes. The accumulation of the executioner of apoptosis, casp3A, in aggresomes limits the execution of apoptosis until its term, and represents an abortosis-like event occurring in Alzheimer’s disease neuronal cells patients. This HSV-1 infected cellular model allowed to uncover novel non-genetically driven cellular and molecular mechanisms linked to the early phases of Alzheimer’s disease development.

## Materials and methods

### Cell lines and virus

B103 murine neuronal cell line is a generous gift of D. Schubert (San Diego-USA). SY-5Y human neuronal cell line is a SY5Y/TR cell line stably transfected with pCDNA Néo with wild-type 695 mRNA APP isoform (APPWT) or not (PC3.1). These cell lines are a generous gift of N. Sergeant. (Lille-FRANCE). All cells were grown as recommended by ATCC.

*Herpes simplex* virus type 1(HSV-1) is the macroplaque strain [18].

#### Infection and treatments

Infection performed in this study (except ELISA analysis) was performed at 1 virus for 1 cell, also called multiplicity of infection of 1 (m.o.i 1). M.o.i is determined from number of cells and virus titer (pfu/mL). For infection, culture medium was removed and the virus diluted in culture medium containing 1% Foetal Bovine Serum (FBS) without antibiotics. The medium was brought into contact with the cells. After 1 h of virus adsorption at 37 °C in a humid atmosphere containing 5% CO2, the medium was removed and the cells were then incubated at 37 °C in culture medium containing 1% FBS without antibiotics until the end of the experiment. Hours post-infection (h.p.i) were determined from the contact of the viral suspension with the cells. A m.o.i of 2 was used for drug treatments at 6 h.p.i (Fig. 6A, B).

Z-VAD-fmk (Calbiochem), nocodazole (Sigma), ALLN (Calbiochem) and Flurbiprofen treatments performed with infection were added to cells 15 h before infection (pre-treatment) and during the course of infection with respective concentrations of 46,7 µg/mL, 20 µg/mL, 10 µg/mL and molarity of 400 µM or 1 mM. Treatment of Flurbiprofen in association with Z-VAD-fmk (Calbiochem) were applied with the same protocol as above with the respective molarity of 400 μM and 100 or 200 μM. All pre-treatments were performed in culture medium containing 10% FBS.

#### Induction of apoptosis by staurosporine

Experiments of apoptosis induced by staurosporine were performed with or without ALLN treatment. A solution at 1 µM was added to cells after or not a pre-treatment by ALLN at a concentration of 10 µg/mL during 15 h.

#### Antibodies

The different antibodies used were:Polyclonal anti-APP C ter antibody (171610-Calbiochem), polyclonal anti-APP C ter antibody (A8717-Sigma), polyclonal anti-Aβ42 C ter antibody which is specific for the cleavage site and requires the presence of free carboxyl group (A1976-Sigma), monoclonal anti-Aβ42 N ter, 6E10, reactive to amino-acid 1–10 of Aβ (SIG-39320-Covance), monoclonal anti-Aβ42 N ter, 4G8, reactive to amino-acid 17–24 of Aβ (SIG-39220-Covance), polyclonal anti-caspase 3 (9662-Cell signaling) antibody, polyclonal anti-caspase 3 cleaved (Asp 175) antibody (9664-Cell signaling), polyclonal anti-Rheb antibody (4935-Cell signaling), polyclonal anti-US11 antibody [18], monoclonal anti-ICP8 antibody (secreted from 39-S hybridoma cells, ATCC N° HB-8180), polyclonal anti-Herpes simplex virus type 1 (B0114-Dako), monoclonal anti γ−tubulin antibody (T6557-sigma), monoclonal anti-vimentin antibody (M0725-Dako).

#### Western-blot

Total proteins were extracted by sample buffers as described previously [19] and electrophoretic separation of protein were performed according to the protocol of Tris-Tricine-SDS-PAGE [20]. For immunoblotting, membranes were blocked for 1h30 at room temperature (RT) with a solution of TBS-T (Tris-HCl 20 mM pH 7,4; NaCl 130 mM; 0.1% of Tween 20) containing 5% of Bovine Serum Albumin (BSA). Then, membranes were blotted with primary antibodies diluted in TBS-T solution containing 2.5% of BSA during 1 h at RT. Membranes were rinsed three times for 10 min in the TBS-T buffer. Primary antibodies were detected by addition of secondary antibodies coupled to HRP-conjugated during 45 min. After three washes with TBS-T buffer, proteins were visualized with Enhanced ChemiLuminescence (ECL) (GE healthcare).

#### Elisa

B103 cells were seeded into a 96 well plate with 50,000 cells per well. 20 h after seeding, B103 cells were infected with HSV-1 at different m.o.i: 0.2; 0.5; 1.5; 4 and 10 viruses per cell. The infection was stopped at 24 h.p.i. The medium was removed and the cells fixed by a glutaraldehyde solution at 1/10 for 15 min at room temperature. The cells were rinsed 3 times with PBS. The first antibody (anti-Herpes simplex virus type 1, Dako) was added for each well at 4.2 mg/L, and incubated for 1 h at room temperature. After 3 washes with PBS, the conjugated HRP-A protein (Bio-Rad) was added at 1/2000 and incubated 1 h at room temperature. The cells were rinsed 3 times with PBS and revealed by the peroxidase substrate (ABST-tablet ROCHE). The optical density (OD) of the colorimetric reaction was measured at 405 nm with a Perkin-Elmer multibell counter.

#### Immunofluorescence

Cells were grown on glass coverslips pre-coated with poly-DL-ornithine at a concentration of 150 mg/L. Cells were fixed with 3% paraformaldehyde (PFA) for 30 min and permeabilized with PBS solution with Triton X100 for 5 min. The cells were rinsed with PBS containing 10 mM of glycine for 10 min. Then, the cells were incubated in PBS containing 25 mM of glycine for 30 min. Coverslips were blocked with antibody buffer (PBS, BSA 0.1 M; NaCl 0.3 M; tween 20 0.5%) + 3% FBS during 2 h. Cells were incubated with primary antibodies diluted in antibody buffer + 1% FBS for 1 h. After 3 washes with PBS containing 10 mM of Glycine, secondary antibodies coupled to fluorochrome Fluoroprobes 488 or 633 diluted in the same buffer were added for 45 min at room temperature. Then, the cells were rinsed 3 times with PBS containing 10 mM of Glycine for 10 min. The Dapi dye was used for nuclei staining. The Fluoromount G (Electron Microscopy Science) was used for mounting coverslips on glass slides. The epifluorescence was visualized with a Zeiss imagerZ1 AXIO straight microscope in conventional microscopy coupled to a Photometrics Coolsnap HQ2 black and white camera and also, with a Zeiss LSM510 microscope coupled to a confocal leica TCS SP2 system. The 3-dimensional reconstruction of the optical sections observed in confocal microscopy was done with the Amira software (Mercury Comp.Systems). For this purpose, optical sections were made every 0.2 μm from the top of the selected cell to the bottom. 3D reconstructions were therefore the compilation of 30 to 42 optical sections according to the selected cells.

When γ tubulin was detected, the cells were fixed by methanol at −20 °C for 6 min, then by 50% methanol-50% acetone at room temperature for 6 min and permeabilized by PFA 4% and 0.5% triton for 15 min at room temperature. Glass coverslips were saturated with 10% BSA for 10 min.

#### RT-qPCR and micro-array

Total RNA was extracted according to the protocol of RNeasy midi kit (Quiagen).

##### RT-qPCR

Retro-transcription of 0.5 µg of total RNA were first performed. SYBR ® Green PCR protocol was performed using a thermocycler light cycler software version 3.5 (Roche). Relative quantification of amplification of *APP* gene transcripts was realized by comparison with amplification of *RPS18* gene. Nucleotide Sequences of primers used for amplify *APP* were:

Forward 5′ GATGTGGGTTCAAACAAAGG 3′

Reverse 3′ CCTACCTACACATGACAAAG 5′

Nucleotide Sequences of primers used for amplify *RPS18* were:

Forward 5′ GACAGAAGGACGTGAAGGATGG 3′

Reverse 3′ CTTGGACACACCCACAGTACG 5′

##### Micro-array

Three or five microgram of total RNA were retro-transcribed and labeled by incorporation of dCTP (α^33^P). ADNc labeled were hybridized on HuSG9k micro-array performed by laboratory « Technologie Avancée pour le Génome et la Clinique (TAGC) » (Marseille-France). Micro-array probe for *APP*:

5′TGTTACCAATCTGAAGTGGGAGCGGCCGCACCAATTTTTTTTTTTTTTTTTTTTTTTTT-3

#### Fluorescence activated cell sorting

Two types of analysis were performed by BD FACS-Canto ^TM^ (Bexton-Dickinson) either (a) the quantification of protein accumulation or (b) quantification of DNA fragmentation.

(a) After determination of threshold from which fluorescence is considered as significantly positive for the two antibodies tested, APP C ter and ICP8, Aβ42 C ter and ICP8 or casp3A and ICP8, cells were distributed in areas named Q1, Q2, Q3 and Q4. Cells distributed in Q1 and Q2 areas are ICP8 positive and cells distributed in Q2 and Q4 area are APP-derived isoforms or Aβ42 C ter or casp3A positive. When we determined number of cells positives for APP-derived isoforms and Aβ42 oligomers accumulation during infection, we selected the Q4 area for NI condition and Q2 + Q4 areas for 9 h.p.i condition. When we also quantified intensity of fluorescence of cells positive for APP-derived isoforms and Aβ42 oligomers accumulation in infected cells, we took into account Q4 area for NI condition and only Q2 area for 9 h.p.i condition. While we quantified intensity of fluorescence of cells positive for Aβ42 oligomers and casp3A accumulation in infected cells treated or not by Z-VAD-fmk or Flurbiprofen and Z-VAD-fmk, we focus the analysis on Q2 area for 9 h.p.i, 9 h.p.i Z-VAD-fmk or 9 h.p.i Flurbiprofen and Z-VAD-fmk conditions. Determination of number of cells positives for casp3A during infection was performed by take into account Q4 area.

(b) Ten thousand cells were analysed. Among these 10 000 cells, we excluded cellular fragments and doublet of cells. We then analysed intensity of fluorescence of DNA labeled by propidium iodide (PI). Cell with intensity of fluorescence about arbitrary value 50 was determined as cell with DNA fragmented represented cells in late stage of apoptosis.

#### Statistics

SAM: Statistical Analysis of Micro-array is dedicated algorithm used for the supervised analysis of variation of transcripts accumulation of genes represented on micro-array. Acquisition and statistical analysis of FACS data was performed following standard procedures [21]. The percentages of positive cells according to different conditions are displayed with 95% confidence intervals. Relative variations compared to the control condition are quantified by relative risk (RR) estimates, and formally tested for the null hypothesis that RR = 1. A statistical difference of positive cells according to the condition is concluded when the *p*-value is < 0.05. When experiments include several replicates, the percentages of positive cells for the different replicates are displayed individually, and the RR are combined using the meta analysis statistical approach under the fixed effect assumption [22].

## Results

### APP processing during HSV-1 infection

We selected the B103 murine neuronal cell line for its ability to exhibit several features similar to that of differentiated neurons [23] (Suppl Fig. 1A). We first showed that B103 can be infected by HSV-1 (Suppl Fig.1B, C) and that infection led to the production of infectious particles (Viral titer was 4.5 10^5^ pfu /mL).

**Fig. 1:**
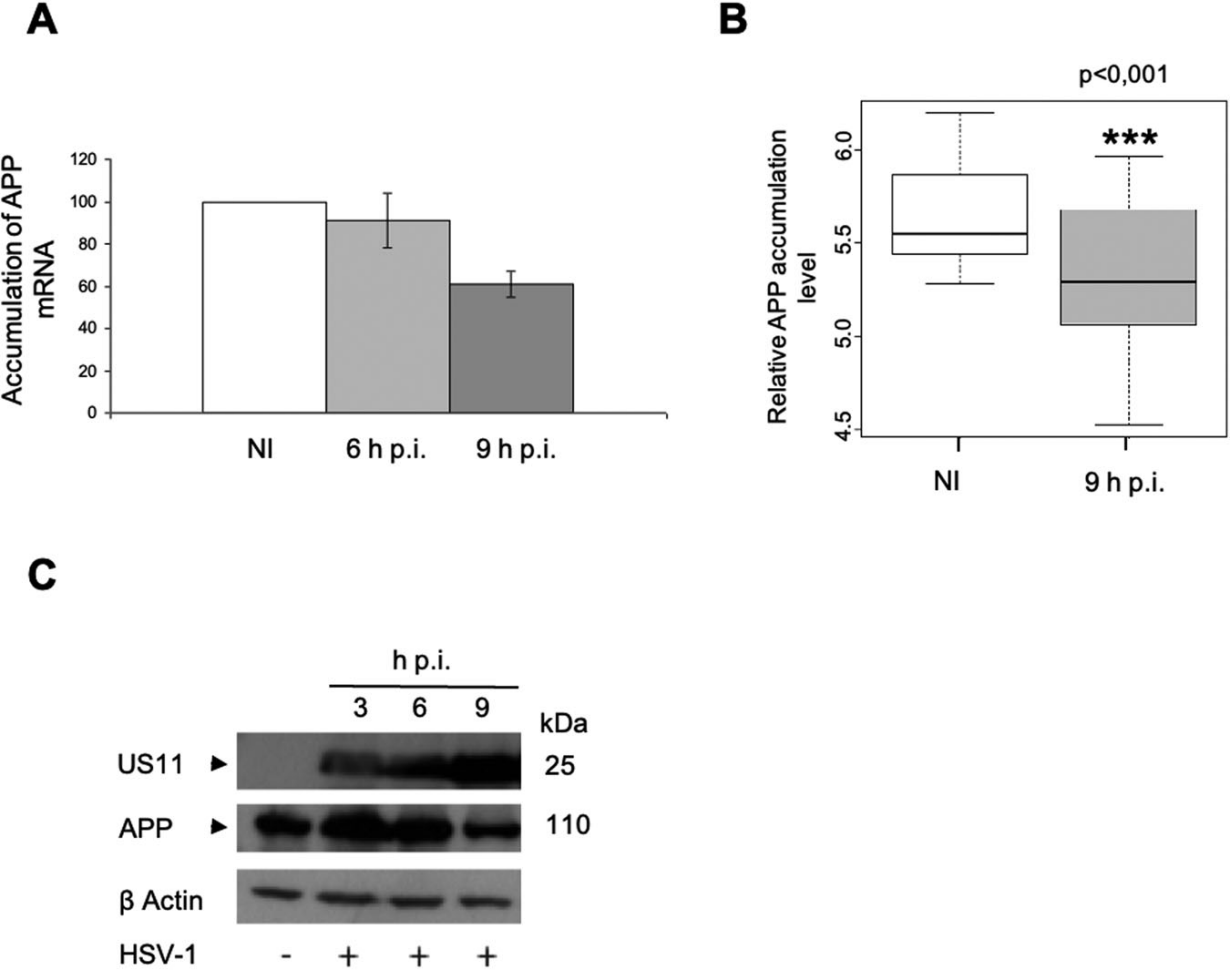
Decreasing of APP amounts during infection. **A** Measurement of accumulation of *APP* mRNA by RT-qPCR at NI, 6 and 9 h p.i with a set of primers recognizing the sequence around the region coding for β amyloid. **B** Box-and-Whisker plot of gene expression measurements for *APP* at NI and 9 h.p.i, *n* = 5, SAM ***p-value < 0.001. **C** WB analysis of APP at NI, 3, 6 and 9 h.p.i using anti-APP Cter antibody (A8717-Sigma). Viral protein US11 as control of infection.

We then determined whether HSV-1 infection led to a modification of amyloid precursor protein (*APP*) gene expression. Quantification of the *APP* transcripts by RT-qPCR showed that their amount decreased progressively during the course of infection (Fig. 1A). A similar decrease was observed by a microarray analysis at 9 h post-infection (h p.i.) (Fig. 1B). Western-blot (WB) detection of APP showed decreased accumulation of the precursor during the course of infection (Fig. 1C). These data indicate that infection induces a decrease of the global amount of APP.

**Fig. 2:**
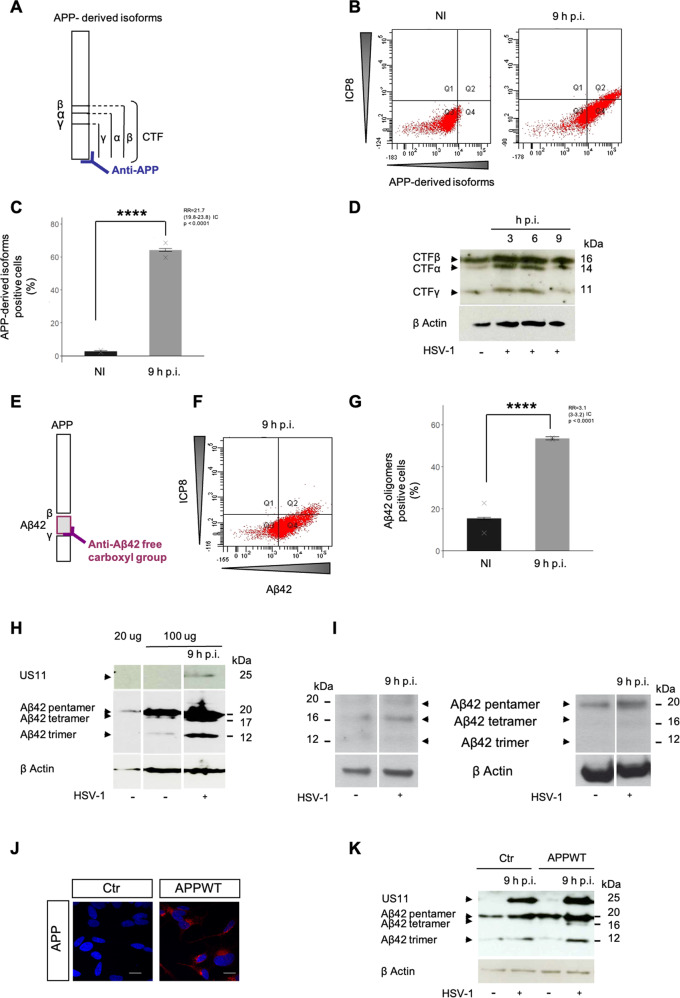
APP post-translational maturation occurs via non amyloid and amyloid pathways during infection. **A** Representation of APP-derived isoforms detected by anti-APP C-Ter antibody directed against the C terminal extremity of APP. **B**, **C** FACS analysis of number of APP-derived isoforms positive cells at NI and 9 h p.i. (%) using anti-APP Cter antibody (171610-Calbiochem) ****p-value < 0.0001 *n* = 1, 2 replicats. **D** WB analysis of APP-derived isoforms at NI, 3, 6 and 9 h.p.i using anti-APP Cter antibody (171610-Calbiochem). **E** Representation of Aβ42 detected by anti-Aβ42 specific antibody directed against C terminal cleaved extremity. **F**, **G** FACS analysis of number of Aβ42 oligomers positive cells at NI and 9 h p.i. (%) using anti-Aβ42 C ter antibody (A1976-Sigma) ****p-value < 0.0001 *n* = 1, 2 replicats. **H** WB analysis of Aβ42 at NI and 9 h p.i. with 20 or 100 µg of proteins using anti- Aβ42 C ter antibody (A1976-Sigma). Viral protein US11 as control of infection. **I** WB analysis of Aβ42 at NI and 9 h p.i. using anti-Aβ42 N ter antibody 6E10 (left panel) or 4G8 (right panel). **J** IF analysis of APP-derived isoforms in SY5Y cell line transfected (APPWT) or not (Control) for human wild type *APP* gene, scale bar = 10 μm. **K** WB analysis of Aβ42 at NI and 9 h p.i. using anti-Aβ42 C ter antibody. Viral protein US11 as control of infection.

Then we have analyzed the effect of infection on APP metabolism. In particular we have characterized the appearance of APP-derived isoforms following infection. It is known that the amyloid pathway leads to the production of a C terminal fragment, CTFβ and to the amyloid peptide, Aβ42 through sequential cleavages by β and γ secretases of the APP. Alternatively, the non-amyloid pathway leads to the production of CTFα, and to a non-amyloid peptide P3 through sequential cleavages by α and γ secretases of APP. Both pathways produce also a common fragment, CTFγ [24]. Considering the APP-derived isoforms positive cell population (Q2 and Q4), fluorescence activated cell sorting (FACS) analysis by anti-APP C-Ter antibody (Fig. 2A) showed a global increase of APP-derived isoforms (Fig. 2B). The increase between non infected (NI) cells and that at 9 h post-infection (h p.i) is about 21 times as indicated by the relative risk value (RR) (Fig. 2C, RR value). Western-blot detection showed that the amount of all the lower molecular weight fragments CTFα, β and γ, increased until 9 h p.i. (Fig. 2D) despite of the decrease of the amount of APP. FACS analysis with anti-Aβ42 C-Ter antibody (Fig. 2E, F) highlighted an increase of Aβ42 accumulation between NI and 9 h p.i. about 3 times (Fig. 2G, RR value). This discrepancy between increase of CTFα, β and γ fragments and Aβ42, confirmed by non overlapped interval of confidence (IC), reveals that infection by HSV-1 activates both pathways. Furthermore, WB analysis performed with a dedicated electrophoretic system highly specific for the fine separation of small peptides (see Methods section) showed an increasing accumulation of Aβ42 oligomeric isoforms during infection of B103 (Fig. 2H). This observation was confirmed with two other antibodies directed against the N terminal part of Aβ42 (Fig. 2I). We also confirmed an increasing accumulation of oligomers of Aβ42 in human neuronal-derived cell line SY5Y transfected (APPWT) or not (Control) with human wild type APP gene (Fig. 2J, K). Altogether these results demonstrate that post-translational processing of APP through both non-amyloid and amyloid pathway is up-activated by HSV-1 infection.

### Aβ42 oligomers production in HSV-1 infected cells

**Fig. 3:**
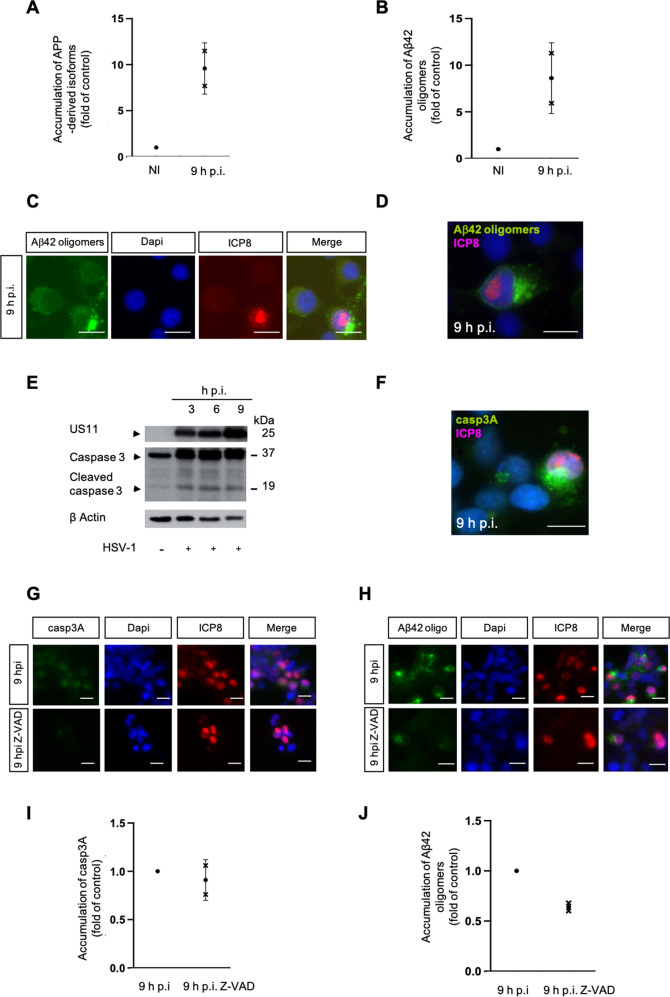
Caspase dependent Aβ42 oligomers production occurs in HSV-1 infected cells. **A** FACS analysis of intensity of fluorescence of APP-derived isoforms positive cells at NI and 9 h p.i. using anti-APP Cter antibody (171610-Calbiochem) Fold of control: 9 h p.i /NI, *n* = 2. **B** FACS analysis of intensity of fluorescence of Aβ42 oligomers positive cells at NI and 9 h p.i. using anti-Aβ42 C ter antibody (A1976-Sigma) Fold of control: 9 h p.i /NI, *n* = 2. **C** IF analyses of Aβ42 oligomers (A1976-Sigma) at NI and 9 h p.i.Viral protein ICP8 as control of infection (magnification X40), scale bar = 10 μm. **D** Higher magnification (X100), scale bar = 10 μm. **E** WB analysis of caspase 3 at NI, 3, 6 and 9 h.p.i. using anti-caspase 3 antibody (9662-Cell signaling). Viral protein US11 as control of infection. **F** IF analyses of casp3A at 9.p.i using anti-casp3 cleaved antibody (9664-Cell signaling). Viral protein ICP8 as control of infection (magnification X100), scale bar = 10 μm. Experimental condition for **G**, **H**, **I** and **J** HSV-1 infection with or without pan-caspase inhibitor Z-VAD-fmk. **G**, **H** IF analysis of cap3A (9664-Cell signaling) and Aβ42 oligomers (A1976-Sigma) at 9 h p.i. Viral protein ICP8 as control of infection (magnification X40), scale bar = 10 μm. **I**, **J** FACS analysis of intensity of fluorescence of casp3A (9664-Cell signaling) and Aβ42 oligomers (A1976-Sigma) positive cells at 9 h p.i and 9 h p.i Z-VAD-fmk. Fold of control: 9 h p.i Z-VAD-fmk /9 h p.i, *n* = 1, 2 replicats.

Because, as expected, efficiency of infection varied from cell to cell, we focused our analysis of APP-derived isoforms and Aβ42 oligomers accumulation in cells expressing a well identified marker of viral infection, the ICP8 viral protein. Considering the HSV-1 infected cells expressing ICP8 (Q2 only), FACS analysis showed that HSV-1 induced increase of APP-derived isoforms between NI and 9 h infected cells about 9 times (Fig. 3A) which was mainly due to an increase of the Aβ42 oligomers presenting an increase about 8 times (Fig. 3B). Immunofluorescence (IF) analyses confirmed this accumulation of Aβ42 oligomers specifically in infected cells (Fig. 3C). Indeed, Aβ42 oligomers were detected only in cells expressing the viral protein ICP8 (Fig. 3D). This result differs from what we have observed previously (Fig. 2C, G) when the entire population was analysed. This indicates that HSV1 induced a shift between the non-amyloid to the amyloid pathway as long as infection progresses. As a consequence, HSV-1 induces the over production of Aβ42 oligomers which accumulates in the cytoplasm.

### Aβ42 oligomers production and apoptosis context

HSV-1 infection induces caspase dependent apoptosis [25]. During apoptosis, production of Aβ is also enhanced by direct or indirect interaction between APP and caspases [19, 26, 27].We therefore determined whether Aβ42 oligomers production was caspase-dependent in HSV-1 infected cells. We observed that the activated form of caspase 3 (casp3A) increased during infection (Fig. 3E) and accumulated in infected cells (Fig. 3F). Accumulation of casp3A (Fig. 3G) and Aβ42 oligomers (Fig. 3H) was abolished by the pan-caspase inhibitor Z-VAD-fmk. These data showed that Aβ42 oligomers production was caspase dependent in HSV-1 infected cells. This result was in accordance with the demonstration of a caspase stimulated production of Aβ by both secretases [19, 28]. Remarkably, the impact of caspases for Aβ42 production in infected cells was then demonstrated by our observation that the decrease of casp3A accumulation was only 9% (Fold of control 0.91 Fig. 3I) in these experimental conditions whereas that of Aβ42 oligomers was 36% (Fold of control 0.64 Fig. 3J). This result showing that a slight decrease of casp3A expression induces a four time decrease of Aβ42 oligomers production is in agreement with those demonstrating that caspase-mediated proteolysis of APP increases the rate of Aβ42 production [27]. Finally, these set of experiments allowed us to propose that HSV-1-induced caspases activation could be responsible of an amplification of Aβ42 production specifically in infected cells.

### Aggresome formation within HSV-1 infected cells

**Fig. 4:**
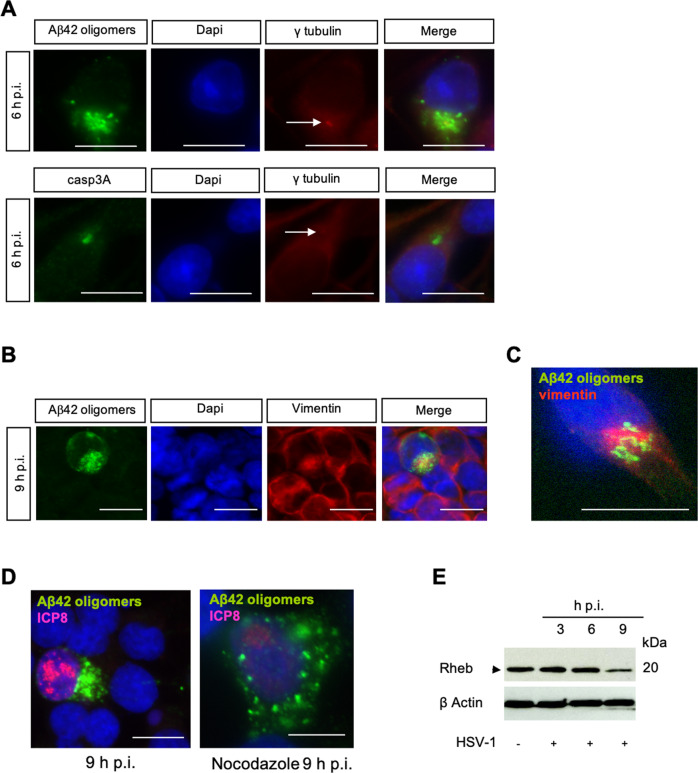
HSV-1 induced accumulation of Aβ42 oligomers and casp3A are aggresome. **A** IF analysis of Aβ42 oligomers (A1976-Sigma) and casp3A (9664-Cell signaling) at 6 h p.i, γ tubulin is a MTOC control (white arrow) (magnification X40), scale bar = 10 μm. **B** IF analysis of Aβ42 oligomers (A1976-Sigma) at 9 h p.i. Vimentin as control of aggresome formation (magnification X40), scale bar = 10 μm. **C** Confocal analysis of colocalization between Aβ42 oligomers and vimentin, scale bar = 10 μm. **D** IF analysis of Aβ42 oligomers (A1976-Sigma) at 9 h p.i with or without nocodazole treatment. Viral protein ICP8 as control of infection (magnification X100), scale bar = 10 μm. **E** WB analysis of Rheb (4935-Cell signaling) at NI, 6 and 9 h p.i using anti-Rheb antibody.

**Fig. 5:**
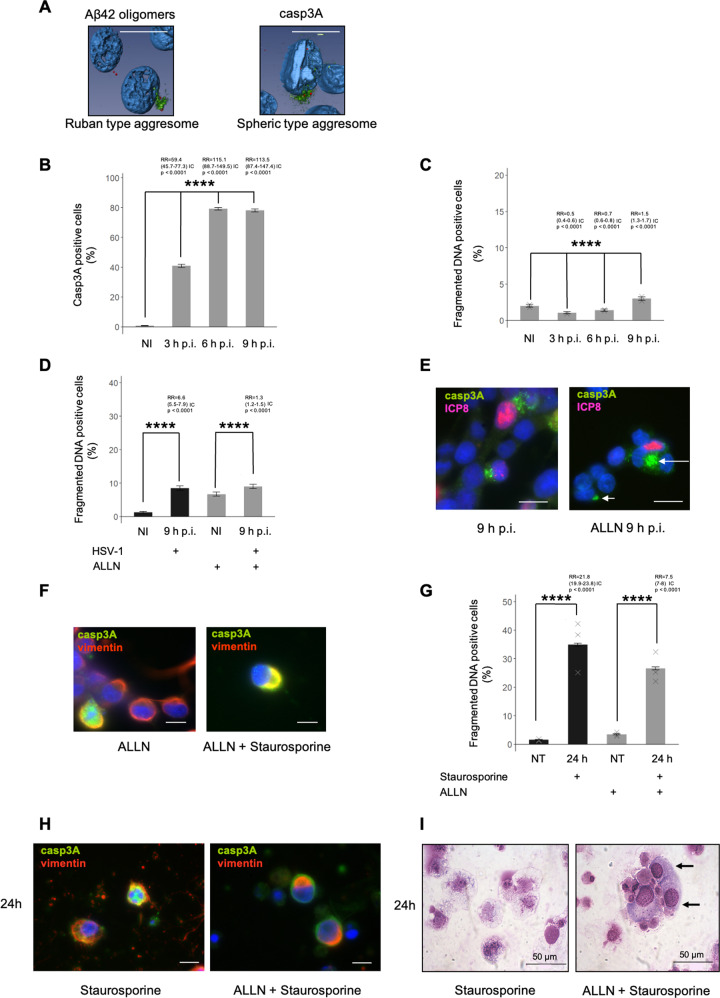
Abortosis-like event by aggresome formation is a cellular mechanism. **A** Characterization of ruban type aggresome of Aβ42 oligomers and spheric type aggresome of casp3A by confocal microscopy analysis and 3D reconstructions. Aβ42 oligomers (A1976-Sigma, green signal), γ tubulin as a MTOC control (red signal), scale bar = 10 μm. **B** FACS analysis of casp3A positive cells using anti-casp3 cleaved antibody (9664-Cell signaling) at NI, 3, 6 and 9 h p.i., ****p-value < 0.0001 *n* = 1. **C** FACS analysis of fragmented DNA positive cells at NI, 3, 6 and 9 h p.i, ****p-value < 0.0001 *n* = 2. Experimental conditions for **D** and **E**: HSV-1 infection with or without ALLN treatment. **D** FACS analysis of fragmented DNA positive cells at NI and 9 h p.i ****p-value < 0.0001 *n* = 1. **E** IF analysis of casp3A (9664-Cell signaling) at 9 h p.i. Viral protein ICP8 as control of infection (magnification X40), scale bar = 10 μm. **F** IF analysis of casp3A (9664-Cell signaling). Vimentine as control of aggresome formation (magnification X100), scale bar = 10 μm. Experimental conditions for **G**, **H** and **I** staurosporine treatment with or without ALLN pre-treatment. **G** FACS analysis of fragmented DNA positive cells at NT and 24 h, ****p-value < 0.0001 *n* = 3. **H** IF analysis of casp3A (9664-Cell signaling) at 24 h post-treatment. Vimentine as control of aggresome formation (magnification X100), scale bar = 10 μm**. I** May Grünwald Giemsa coloration of cells (magnification X50) at 24 h post-treatment, scale bar = 50 μm. Cells without apoptotic phenotype indicated by black arrows.

While performing the experiments described above we noticed that cytoplasmic accumulations of Aβ42 oligomers and casp3A were always perinuclear and asymmetric suggesting that they accumulate within subcellular structures called aggresomes. Aggresomes, observed in Alzheimer’s disease cells in condition of proteotoxic stress [29–31], represent the dynamic recruitment of unfolded proteins via the microtubule network to the microtubule organizing center (MTOC) where vimentin as a “cage” takes place around these aggregated proteins [32–34]. This process has been described as a way to store misfolded proteins when proteasome is inhibited or overwhelmed. We therefore wanted to characterize if the peptides and proteins found in infected cells were aggregated in these virally-induced aggresomes. Confocal microscopy coupled to 3D reconstructions revealed that Aβ42 oligomers and casp3A aggregated at the MTOC (white arrows) (Fig. 4A) adopting two different types of aggresomes’ structures described previously [35] (Fig. 5A). IF followed by confocal analysis confirm that Aβ42 oligomers aggregates were surrounded by vimentin (Fig. 4B, C) and disappeared from the MTOC when destabilization of microtubules network was obtained by nocodazole [36] treatment during infection (Fig. 4D). A correlation between aggresome formation and the down-regulation of Rheb accumulation was observed at 9 h p.i (Fig. 4E). Indeed, it has been demonstrated that regulation of aggresome formation was a Rheb dependent mechanism involving the TSC-Rheb-mTOR pathway [37]. It has been shown that a high Rheb activity inhibits aggresome formation by disrupting interaction between the unfolded proteins and the microtubule network, thus limiting their transport to the MTOC. Collectively, these findings demonstrate that Aβ42 oligomers and casp3A are aggregated in HSV-1 induced aggresomes.

### Casp3A sequestration in aggresome and final stage of apoptosis

FACS analyses showed that, casp3A accumulated during the course of infection (Fig. 5B) while the late stage of apoptosis failed to occur (Fig. 5C). We therefore determined whether this localization could represent a sequestration of the active form of casp3 and as consequence could affect its cellular function as the final executor of apoptosis. We used an inhibitor of proteasome ALLN [32] to promote casp3A aggresome accumulation during infection. We quantified an increase of about 1.3 fold (RR value) of apoptotic cells in the late stage of apoptosis (fragmented DNA) in opposition to 6.6 (RR value) for an infection without treatment (Fig. 5D). By IF, we showed that casp3A-containing aggresome formation was amplified during infection with ALLN (white arrows –right panel) (Fig. 5E). We then evaluated the possibility that casp3A-containing aggresome formation could be a cellular mechanism occurring during apoptosis and precluding its execution. For this, aggresome formation was induced by ALLN pre-treatment and casp3A expression was obtained by a treatment with staurosporine which is known to induce caspase-dependent apoptosis [38] (Fig. 5F). Efficiency of apoptosis was then evaluated by measuring the percentage of cells in late stage of the apoptotic process. As expected, 24 h of staurosporine treatment induces an increase of cells in late stage of apoptosis of about 22 times (RR value) (Fig. 5G). However, when cells have been first pre-treated with ALLN this increase is only 7.5 times (RR value) showing a significative limitation of fragmented DNA cells. Presence of aggresomes - confirmed by IF analyses (Fig. 5)—is associated to this strong impairement of the progression of the apoptotic process. Moreover, an enrichment of cells with non-apoptotic phenotype when staurosporine was used after ALLN treatment is shown in Fig. 5I (black arrows –right panel). In addition, the observation that the percentage of cells in early stage of apoptosis (annexin V positive cells) was higher after staurosporine treatment in cells pre-treated with ALLN than in cells non pre-treated (not shown) supported the notion that aggresome formation inhibits apoptosis execution but not apoptosis initiation. Therefore altogether, these data showed that sequestration of the active form of casp3 within aggresome precludes the final step of apoptosis.

### NSAIDs like flurbiprofen and Aβ42 oligomers production

**Fig. 6:**
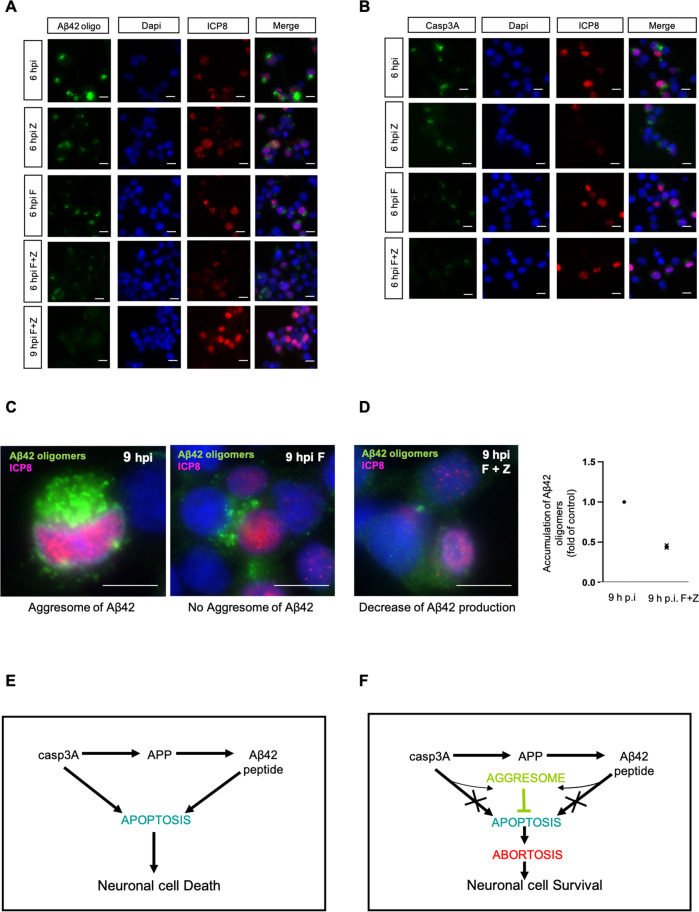
Flurbiprofen associated to anti-apoptotic molecule abrogate Aβ42 oligomers production in HSV-1 infected cells. Experimental conditions for **A**, **B**, **C** and **D**: HSV-1 infection with or without Z-VAD-fmk (Z), Flurbiprofen (F) and Flubriprofen (F) associated to Z-VAD-fmk (Z) treatments. **A** IF analysis of Aβ42 at 6 h p.i. and 9 h p.i. using anti-Aβ42 C ter antibody (A1976-Sigma). Viral protein ICP8 as control of infection (magnification X40), scale bar = 10 μm**. B** IF analysis of casp3A at 6 h p.i. using anti-casp3 cleaved antibody (9664-Cell signaling). Viral protein ICP8 as control of infection (magnification X40), scale bar = 10 μm. **C** IF analysis of Aβ42 (A1976-Sigma) at 9 h p.i. and 9 h p.i. with Flurbiprofen, viral protein ICP8 as control of infection (magnification X100), scale bar = 10 μm. **D** IF analysis of Aβ42 (A1976-Sigma) at 9 h p.i. with Flurbiprofen and Z-VAD-fmk, viral protein ICP8 as control of infection (magnification X100), scale bar = 10 μm (left panel). FACS analysis of intensity of fluorescence of Aβ42 oligomers positive cells (A1976-Sigma) at 9 h p.i and 9 h p.i with Flurbiprofen and Z-VAD-fmk. Fold of control: 9 h p.i Flurbiprofen and Z-VAD-fmk /9 h p.i., *n* = 1, 2 replicats (right panel). **E** HSV-1 induces the appearance of activated caspase 3 (casp3A) resulting in caspase-dependent cleavage of APP. This leads to the production of Aβ42 peptides. Casp3A and Aβ42 are two pro-apoptotic factors that should induce apoptosis and therefore neuronal death. **F** However, HSV-1 induces simultaneously to the production of casp3A and Aβ42 peptides, the formation of intra-cytoplasmic aggresomes in which these two factors accumulate. This cytoplasmic sequestration precludes apoptosis to proceed until late stages. This phenomenon, called abortosis allows neuronal cell survival and a chronic production of Aβ42.

Because we showed an interplay between casp3 activation and Aβ42 oligomers production, we tested the NSAID flurbiprofen described as potential anti-Alzheimer’s disease drug [39] in combination with a pan caspase inhibitor, Z-VAD-fmk. As shown in Fig. 6A, Flurbiprofen impaired Aβ42 oligomers production as that observed for the pan-caspase inhibitor Z-VAD-fmk used as a positive control. Moreover, when Flurbiprofen and Z-VAD-fmk were associated, we observed a synergistic effect inducing a strong limitation of Aβ42 oligomers accumulation at 6 h.p.i and a complete abrogation at 9 h.p.i. IF analysis also showed that Flurbiprofen and/or Z-VAD-fmk decreased casp3A expression suggesting that the effects on Aβ42 oligomers accumulation were due to their capability to reduce apoptosis context (Fig. 6B). Furthermore, as shown in Fig. 6C flurbiprofen inhibited aggresomes formation. In consequences, association of flurbiprofen with Z-VAD-fmk strongly limits Aβ42 oligomers production and aggregation as demonstrated by IF (Fig. 6D, left panel). Decrease of Aβ42 oligomers accumulation was confirmed by FACS analysis (Fig. 6D, right panel). Collectively, these results reinforce the crucial impact of the apoptotic context on Aβ42 oligomers production and confirm Flurbiprofen as promising drug for treatment of Alzheimer’s disease.

## Discussion

To illustrate the interplay between HSV-1 and the host neuronal cells potentially involved in chronical pathology such as Alzheimer’s disease, we have analyzed the modifications of the wild type APP metabolism induced by infection. We have pointed out a modification of the APP post-translational maturation during the course of infection leading to a production of APP-derived peptides preferentially through activation of the amyloid pathway. We have first demonstrated that HSV-1 induces an increase of oligomers of Aβ42. We have shown that this increase in Aβ42 is specific of the infected neuronal cells. Indeed, the production of Aβ42 in infected cells is casp3A dependent. Importantly because caspase 3 activation [40] and caspase dependent production of Aβ [19, 41] are two of the molecular events taking place in the brain of Alzheimer’s disease patients it is possible that HSV-1, during the reactivation phases, by stimulating these processes participates to Alzheimer’s disease initiation and/or progression of subjects chronically infected by this virus.

We have shown that Aβ42 but also casp3A accumulates within intracytoplasmic structures that we have clearly identified as aggresomes. Interestingly, other studies have highlighted an intracellular accumulation of Aβ42 as a first step before the extracellular amyloid plaque formation in brain of Alzheimer’s disease patients and animal models [42–45]. In addition, the HSV-1 induced aggresomes are very similar to the cytoplasmic regions that have been described close to the nuclear envelope of pyramidal cells of Alzheimer’s disease brain patients and that were shown to contain large amounts of Aβ42 [46]. This suggests that HSV-1, by inducing Aβ42 cytoplasmic accumulation could potentially participate to early stages of Alzheimer’s disease process in infected patients. Furthermore, as we showed that this accumulation is dependent of the casp3 activation, as previously shown by Gervais et al. [27] and that the two proteins are accumulated in aggresomes, we could propose that formation of aggresomes in which casp3A or Aβ42 oligomers accumulates are molecular mechanisms participating to the early phase of the disease. Therefore, any molecules that may destabilize aggresome formation and/or limit the expression of casp3A like flurbiprofen in synergistic association with pan-caspase inhibitor Z-VAD-fmk should abrogate Aβ42 oligomers production/aggregation and in consequences the course of the disease when administrated early. This is in accordance with the extended analysis of anti-inflammatory prevention trial (ADAPT) demonstrating that NSAIDs like flurbiprofen have diverse effects depending on the stage of the disease. Indeed, while treatment could have an adverse effect in the latter stage of the Alzheimer’s disease pathogenesis, its benefit was effective in early stage of the disease limiting Alzheimer’s disease incidence when asymptomatic individuals were treated during 2–3 years [47].

Finally, we have shown that sequestration of casp3A within aggresomes but also of Aβ42 which exhibits pro-apoptotic functions strongly inhibits both virally and drug-induced apoptosis. This result is reminiscent to a phenomenon observed in Alzheimer’s disease called abortosis [48]. Abortosis is characterized by induction of apoptosis without observation of features of late stages of apoptosis (condensation of chromatine, nuclear segmentation, destructuration of membrane and apoptotics bodies formation) due to a lack of distal transmission of the caspase-mediated apoptotic signal [49]. Indeed, some neurones of Alzheimer’s disease patients exhibit markers of apoptosis such as casp3A [50, 40] without final apoptotic characteristics [49]. Consequently infection of neuronal cells by HSV-1, that leads to the formation of aggresomes in which casp3A or Aβ42 accumulates, could induce an amplification of intracellular Aβ42 deposition and aggregation and provide a kind of amplification loop allowing a continuous production of Aβ42, however, without neuronal death, in neurons of patients chronically infected by HSV-1 (Fig. 6E, F). These infected viable neurons could therefore provide a continuous source of Aβ42 production. It can be proposed that caspase-dependent production of Aβ42 oligomers [27] in this context of abortosis-like event represents a “vicious circle” leading to the chronic amplification of the Aβ42 intracellular accumulation and aggregation that participate to the establishment of degenerative disorder like Alzheimer’s disease in patients infected by HSV-1. This hypothesis is supported by the fact that this HSV-1 induced phenotype is extremely similar to the one observed in neurons of patients in the early stages of Alzheimer’s disease.

Collectively our findings suggest that formation of aggresomes containing pro-apoptotic factors such as casp3A [51] or intracellular Aβ42 [52] could be a cellular mechanism for neuronal cells to escape apoptosis induce by external stresses and hijacked by HSV-1, as described for numerous viral determinants encoded [53], in order to favor its persistence in neuronal cells. In conclusion, these cascade of molecular events, in addition to the epidemiological studies support the notion that HSV-1 could acts as a chronic molecular co-factor of Alzheimer’s disease by engaging neurons in the early molecular steps of the disease, inducing caspase-dependent production and aggregation of Aβ42 oligomers in aggresome and stabilizing them in an abortosis-like state.

## Supplementary information

supplemental Figure 1
