# Supplementary material for: HSV-1 cellular model reveals links between aggresome formation and early step of Alzheimer’s disease
Source: Transl Psychiatry. 2023 Mar 10;13:86. doi: 10.1038/s41398-023-02376-8 (PMC10006237; doi:10.1038/s41398-023-02376-8)
Supplement: Supplementary file 1 — supplemental Figure 1 [file 41398_2023_2376_MOESM1_ESM.docx]

# Supplementary Figure


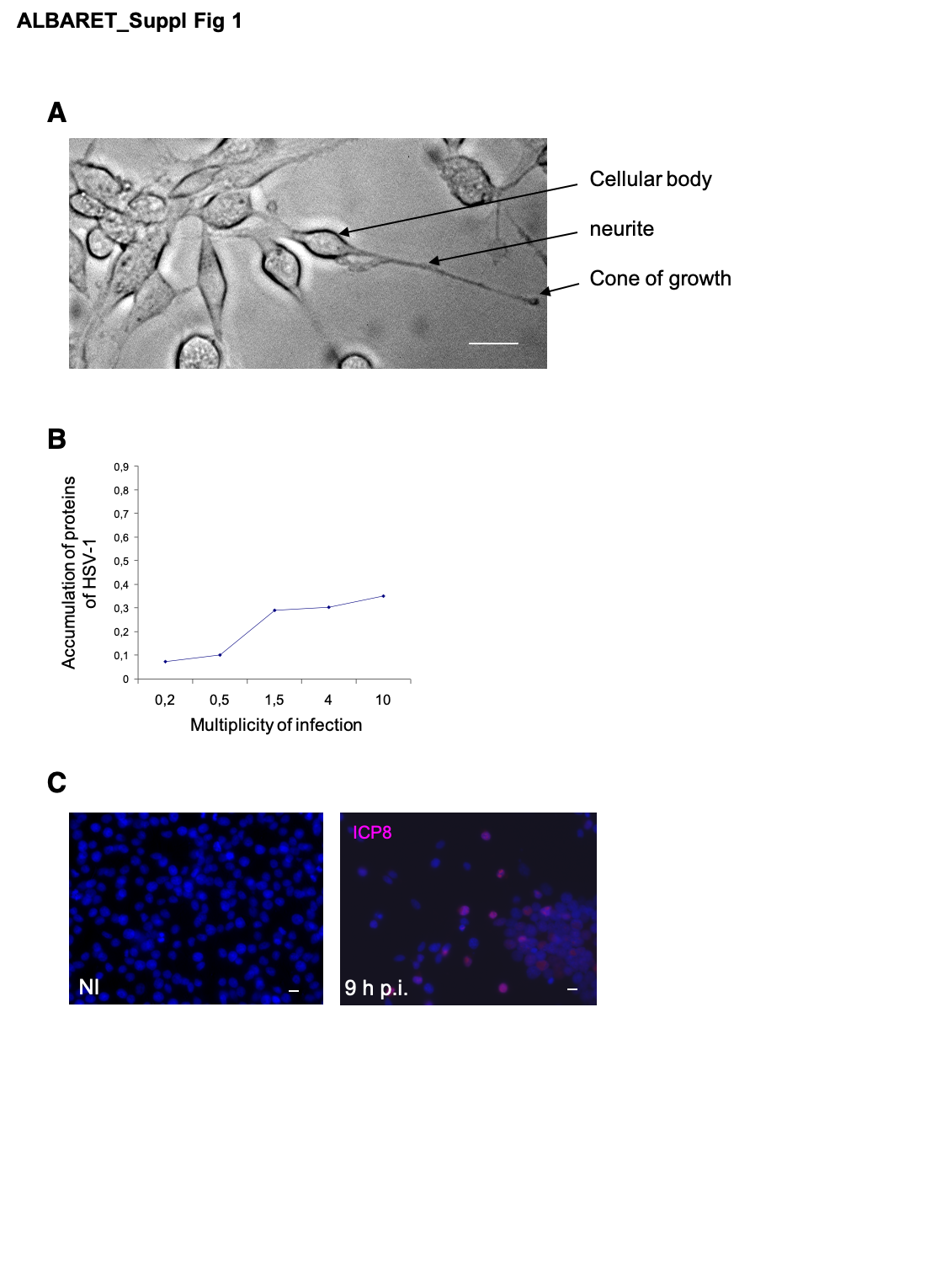


**Suppl Fig. 1. Productive infection of B103 with HSV-1**

**A**, B103 cells in culture. Phase contrast (magnification X40), scale bar = 10 μm. **B**, Quantification of HSV-1 protein by ELISA at 24 h p.i. of an infection from 0.2 up to 10 multiplicity of infection (m.o.i.) **C**, IF analysis of ICP8 viral protein by immunofluorescence at NI and 9 h p.i, scale bar = 10 μm.
